# Bacterial Nosocomial Infections and Antimicrobial Susceptibility Pattern among Patients Admitted at Hiwot Fana Specialized University Hospital, Eastern Ethiopia

**DOI:** 10.1155/2018/2127814

**Published:** 2018-12-04

**Authors:** Moti Tolera, Degu Abate, Merga Dheresa, Dadi Marami

**Affiliations:** ^1^School of Public Health, College of Health and Medical Sciences, Haramaya University, P.O.B: 235, Harar, Ethiopia; ^2^Department of Medical Laboratory Sciences, College of Health and Medical Sciences, Haramaya University, P.O.B: 235, Harar, Ethiopia; ^3^School of Nursing and Midwifery, College of Health and Medical Sciences, Haramaya University, P.O.B: 235, Harar, Ethiopia

## Abstract

Nosocomial infections remain a major cause of mortality and morbidity worldwide. Despite the highly specialized interventions and policies, the rate of infection is still high due to the emergence of antimicrobial-resistant bacteria. This study described the prevalence of bacterial nosocomial infections and antimicrobial susceptibility pattern of isolates among patients admitted at Hiwot Fana Specialized University Hospital, Eastern Ethiopia. A hospital-based cross-sectional study was conducted among 394 nosocomial infection-suspected patients from March 2017 to July 2017. Data were collected using a structured questionnaire. Specimens from the respective site of infections were collected and examined for the presence of pathogenic bacteria and their antimicrobial susceptibility using standard culture and serological tests. Data were summarized using descriptive statistics. The prevalence of culture-confirmed bacterial nosocomial infection was 6.9% (95% CI: 4.3–7.9). *Staphylococcus aureus* (18.5%) was the most common isolate followed by *Escherichia coli* (16.7%). *S. aureus* showed 80% resistance to chloramphenicol and erythromycin, and 70% to cephalexin and tetracycline, respectively. A methicillin-resistant *S. aureus* made up 88.9% of all *S. aureus* isolates. *Pseudomonas aeruginosa* showed 83.7% resistance to each of ceftazidime and cephalexin, and 66.7% to chloramphenicol. The most common multidrug-resistant isolates were *P. aeruginosa* (30.4%) and *S. aureus* (21.7%). The prevalence of nosocomial infections in this study was comparable with other findings; however, the high rates of antimicrobial resistant isolates represent a substantial threat to the patients, communities, health care providers, and modern medical practices. Bacterial nosocomial infection treatment should be supported by culture isolation and antimicrobial susceptibility testing.

## 1. Introduction

A nosocomial infection (NI) (also known as hospital-acquired infection) is a localized or a systemic infection resulting from an adverse reaction to infectious agents or its toxins that develops in 48 hours or more after admission and was not incubating on admission [1, 2]. The most common type of NIs are urinary tract infections (usually catheter associated) (31%) [3] followed by surgical site infections (SSIs) (17%), primary bloodstream infections (BSIs) (usually associated with the use of an intravascular device) (14%), and pneumonia (usually ventilator associated) (13%) [3, 4]. The main bacteria associated with NIs are *S. aureus*, coagulase-negative staphylococci (CoNS), *Streptococcus pneumoniae*, *Escherichia coli*, *P. aeruginosa, Haemophilus influenzae, Klebsiella pneumoniae, Acinetobacter*, and *Enterococci* [5, 6]. The transmission within the hospital occurs through cross-contamination of the patients via the contaminated hands of health care staffs who come in frequent contact with patients or through contaminated objects [4, 7].

The emergence of antimicrobial-resistant bacteria has become a public health problem, creating a new burden on modern medical care in hospitals [8, 9]. *P. aeruginosa*, *S. aureus*, *Enterococci* spp., *Klebsiella* spp., and *Enterobacter* spp. are the major resistant pathogens of concern [10, 11]. Particularly, methicillin-resistant *S. aureus* (MRSA) is known to cause considerable morbidity and mortality in hospitalized patients [12, 13]. The consequence of the infection caused by resistant bacteria lies in their ability to not only alter the outcome of critically ill patients but also reduce the chances of the treatment, prolong the duration of the hospitalization, increase the cost of health care, and make the spread of infection easier and the prevention more difficult [14, 15].

Knowledge of proper antimicrobial prescription policy of a particular setting is crucial to optimize the management and reduction of the rate of NIs; however, the investigation of causative agents and their antimicrobial susceptibility profile are an essential prerequisite [16, 17]. In Ethiopia, some comprehensive studies were conducted on NIs [18–20]; none of these include most of the sources of NIs and have not determined the antimicrobial susceptibility pattern of the causative agents. Moreover, a study reported in one region is not necessarily reflecting the status of other regions. This study was carried out to assess the prevalence and antimicrobial susceptibility pattern of bacteria causing NIs among patients admitted at Hiwot Fana Specialized University Hospital, Eastern Ethiopia.

## 2. Materials and Methods

### 2.1. Study Setting and Design

A cross-sectional quantitative study involving bacteriological analysis was conducted at Hiwot Fana Specialized University Hospital, Harar, Eastern Ethiopia from March 2017 to July 2017. Harar is the political and administrative town of the Harari Regional State and is located at 525 km from Addis Ababa, Ethiopia. There are six hospitals and eight health centers in this region. Hiwot Fana Specialized University Hospital provides health care services and serve as a referral hospital for eastern parts of our country. It has the largest client load with an average bed occupancy rate of 83% (sources: Hiwot Fana Specialized University Hospital Annual Report of 2016). The hospital consists of six major wards: Medical, Surgical, Obstetrics, Gynecology, Malnutrition, and Pediatric wards.

### 2.2. Study Population

Patients admitted to the Medical, Surgical, Obstetrics, Gynecology, Malnutrition, and Pediatric wards for more than 48 hours and who had a clinical evidence of NIs were included in this study.

### 2.3. Sample Size and Sampling Technique

A single population proportion formula was used to calculate a sample size, assuming 95% confidence level, 3% margin of error, 10% predicted nonresponse rate, and 10.3% prevalence of NIs [1]. The final sample size was determined to be 433. The study participants were selected consecutively until the required sample size fulfilled.

### 2.4. Data and Specimen Collection

Patients admitted in Medical, Surgical, Obstetrics, Gynecology, Malnutrition, and Pediatric wards were followed prospectively for the development of NIs by the clinicians. The patients were assessed for SSIs, UTIs, respiratory infections, and BSIs as per the Center for Disease Control and Prevention (CDC) criteria [21]. Data were collected from selected patients using a questionnaire by five trained nurses. A questionnaire was developed from different kinds of literature [4, 5, 20].

Clinical specimens such as a clean-catch midstream urine, blood, wound swab, throat swab, nasal swab, and other body fluids were collected by trained laboratory technologists using standard procedures as described by Horan et al. [22] from the respective site of infections. The specimens were labeled with the patient's identification, packed and transported to the Bacteriology Laboratory of the Department of Medical Laboratory Sciences, College of Health and Medical Sciences, Haramaya University, within 30 minutes of the collection in a cold box for analysis.

### 2.5. Phenotypic Characterization of the Isolates

In vitro phenotypic characterization of bacteria was carried out using standard culture and biochemical tests as described by Cheesbrough [23]. In brief, each specimen was streaked onto differential and selective culture media (Oxoid, Ltd., UK), including MacConkey agar, cystine lactose electrolyte-deficient agar, mannitol salt agar, Pseudosel agar (cetrimide agar), and 5% sheep blood agar, for investigation of bacteria. The bacterial isolates were characterized based on colonial morphology, pigmentation of the colony, and cell morphology. Further characterization of the isolates to the species level was performed using biochemical tests, including coagulase, catalase, oxidase, urease, indole, citrate, glucose and lactose fermentation, triple sugar iron agar, bile solubility, and motility tests. Reading of the culture was done by two senior medical microbiologists after overnight incubation at 37°C. UTI was defined when patients had a fever (>38°C), urgency, frequency, dysuria, or suprapubic tenderness and a positive urine culture, i.e., ≥10^5^ colonies forming unit per ml of urine with no more than two species of bacteria [22].

### 2.6. Antimicrobial Susceptibility Testing

A modified Kirby Bauer disk diffusion method was used to test each isolate for in vitro antimicrobial susceptibility based on the Clinical and Laboratory Standards Institute criteria [24]. In brief, standard inoculum adjusted to 0.5 McFarland standard turbidity was uniformly distributed over the surface of Mueller Hinton agar (Oxoid, Ltd., UK). Antimicrobial disks, including (Oxoid, Ltd., UK) cephalexin (30 *μ*g), ceftazidime (30 *μ*g), ceftriaxone (30 *μ*g), chloramphenicol (30 *μ*g), erythromycin (15 *μ*g), gentamycin (10 *μ*g), penicillin (10 *μ*g), tetracycline (30 *μ*g), and cefoxitin (10 *μ*g), were applied on Mueller Hinton agar plates using automatic disk dispenser. Following overnight incubation at 37°C, the zone of inhibition was measured and interpreted as sensitive, intermediate sensitive or resistant per the standard criteria [24]. Methicillin resistance was confirmed for all cefoxitin-resistant *S. aureus* using a PBP2a-agglutination test as described by Atay and Gulay [25]. An isolate was considered multidrug resistant (MDR) if it is resistant to at least one agent in three or more antimicrobials of structurally different categories [26].

### 2.7. Data Quality Control

The questionnaire was initially prepared in English and translated into local languages (*Amharic* and *Afan Oromo*) by a language expert and back to English by another language expert to check the consistency. The questionnaire was pretested on 5% NI-suspected patients in the Dil Chora Referral Hospital, Dire Dawa, Ethiopia, before data collection to check its simplicity. Data collectors were recruited from other hospitals in the region and trained for 4 days on the data collection procedure and interview techniques to minimize bias. Universal precautions were strictly followed during specimen collection, culture preparation, inoculation, and isolation. The sterility of the newly opened medium was checked before use. Recommended reference strains, including *E. coli* (ATCC® 25922), *K. pneumoniae* (ATCC® 700603), *S. aureus* (ATCC® 25923), *S. pneumoniae* (ATCC® 49619), and *P. aeruginosa* (ATCC® 27853), were used to check the performance of each medium and antimicrobial disks.

### 2.8. Method of Data Analysis

Data were entered into the EpiData software (v. 2; Odense, Denmark) and analyzed using the Statistical Package for Social Sciences software (v. 22; SPSS, Inc, Illinois, USA). The prevalence of NIs was presented in percentage along with the 95% confidence interval (CI). Two or more bacteria isolated from one patient were categorized as one bacterium for summarizing the prevalence but were analyzed separately for the antimicrobial susceptibility profile. Each bacterium was tested in triplicate for a single antimicrobial, and the mean value was taken to determine its antimicrobial susceptibility pattern. Intermediate results were included in the resistant category for analysis.

### 2.9. Ethical Consideration

The study was reviewed and ethically approved by the Institutional Health Research Ethics Review Committee of the College of Health and Medical Sciences, Haramaya University. Informed, voluntary, written, and signed consent/assent was obtained from each parent/caretaker or participant. Positive finding was communicated to attending clinician for appropriate treatment.

## 3. Results

### 3.1. Patient Characteristics and Prevalence of NIs

A total of 394 clinically suspected patients for NIs were included in this study. The majority of study participants were females, 223 (56.6%), with a male-to-female ratio of 0.8 : 1. The mean age of participants was 23.9 years (±18.3 standard deviation). A large number of participants were admitted to Obstetrics/Gynecology (26.1%) followed by Medical ward (25.9%). The majority (86.3%) of patients had no previous history of admission. The length of stay of the patients on the admission was 4–7 days (37.3%) (Table 1).

The overall prevalence of culture-confirmed NIs was 6.9% (95% CI: 4.3–7.9). A total of 54 bacterial pathogens were recovered. Of these, 30 (55.6%) were Gram-positive bacteria. The most common bacteria were *S. aureus* (18.5%) followed by *E. coli* (16.7%) and *S. pneumoniae* (14.8%). Surgical sites were most frequently infected (31.5%) followed by the bloodstream (25.9%). *S. aureus* (29.4%), *P. aeruginosa* (17.6%), and CoNS (17.6%) were the most common types of pathogen isolated from surgical sites, while *E. coli* (36.3%), *Proteus* spp. (18.2%), and *Enterococcus* spp. (18.2%) were from urinary tract. *S. pneumoniae* (41.6%) and *Klebsiella* spp. (25%) were the top two pathogens isolated from the upper respiratory tract. The most frequently isolated bacteria from the bloodstream were *S. aureus* (28.6%), *E. coli* (21.4%), and *S. pneumoniae* (21.4%) (Table 2).

### 3.2. Antimicrobial Susceptibility Pattern

Of the total Gram-positive bacteria, 73.3% were resistant to tetracycline, 66.7% to erythromycin, and 53.3% to penicillin, while all other isolates showed sensitivity to antimicrobials in the testing panel. Of the Gram-positive bacteria*, S. aureus* showed resistance to a number of antimicrobials (80% resistance to each of chloramphenicol and erythromycin, 70% to cephalexin and tetracycline). *S. pneumoniae* isolates were 75% resistant to each of tetracycline and erythromycin, and 62.5% to penicillin. CoNS were 60% resistance to each of erythromycin and tetracycline. *Streptococcus* spp. showed 100% resistance to each of cephalexin, erythromycin, and tetracycline. MRSA accounted for 88.9% of a total number of *S. aureus* isolates (Table 3).

All Gram-negative isolates were found to be resistant to ceftazidime (66.7%) and chloramphenicol (66.5%), while showed sensitivity to ciprofloxacin (78.5%), ceftriaxone (70.8%), gentamycin (66.7%), and cephalexin (54.2%). The most resistant to a number of antimicrobials was *P. aeruginosa*, which showed 83.7% resistance to each of ceftazidime and cephalexin, and 66.7% to chloramphenicol. *E. coli* were resistant to ceftazidime (77.8%) and chloramphenicol (66.7%). *Klebsiella* spp. exhibited 75% resistance to ceftazidime. *P. vulgaris* showed 66.7% resistance to chloramphenicol (Table 4).

### 3.3. Multidrug Resistance

**Figure 1:**
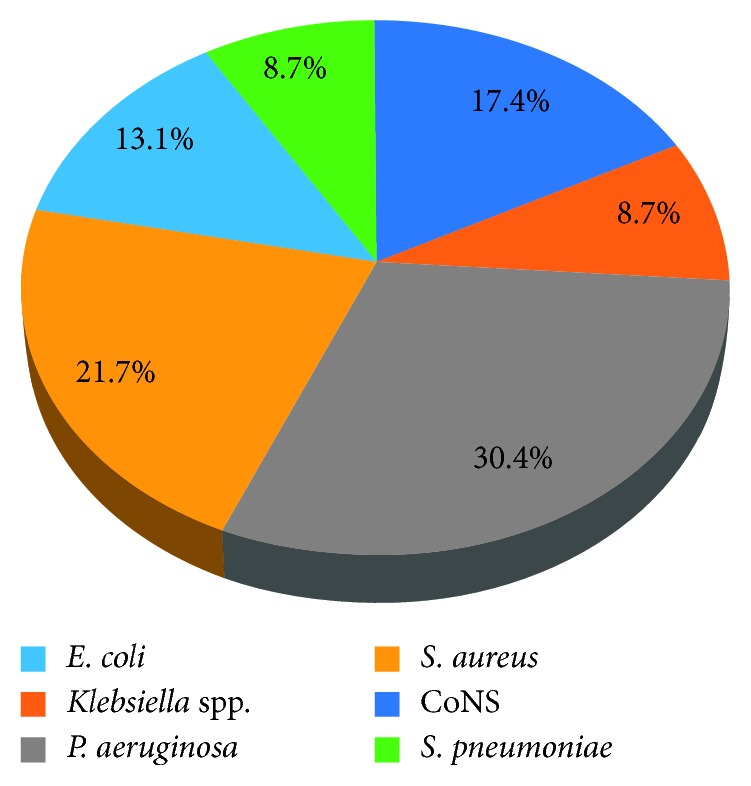
Multidrug resistant bacteria isolated from NI-suspected patients at Hiwot Fana Specialized University Hospital, Eastern Ethiopia, March 2017 to July 2017.

The overall prevalence of multidrug resistance (MDR) bacteria was 42.6% (23/54). The predominant MDR bacteria were *P. aeruginosa* (30.4%) and *S. aureus* (21.7%) (Figure 1).

## 4. Discussion

Nosocomial infections are one of the major public health problems around the world that vary from one country to the other. The prevalence of culture-confirmed NIs in this study was 6.9% (95% CI: 4.3–7.9). This was lower compared with a previous study conducted in a Tertiary Care Hospital, Ethiopia (35.8%) [20], and Rabat, Morocco (10.3%) [1], but higher compared to the study reported in Lambarene, Gabon (0.3%) [27], and Mazandaran, India (1.03%) [5]. The higher prevalence of NIs in the present study might be due to the inclusion of all age groups and a large number of different kinds of specimens from different wards; the other studies only looked at adult patients and focused on limited types of specimens.

The prevalence of SSIs (31.5%) in this study was higher compared with a study reported from Bahirdar, Ethiopia (10.9%) [19], and Zahedan, Iran (8.6%) [14]. The higher prevalence of SSIs in this study could be explained by the lack of appropriate institutional strategies for the prevention and control of bacterial infections. It could also be due to the fact that this hospital services as a referral hospital that receives patients with severe injury (Mencha injury) from all over the eastern part of our country, which facilitates the circulation of pathogenic bacterial strains that cause SSIs.

The prevalence of BSIs (25.9%) in this study was relatively comparable with the findings of a study conducted in Liaquat University Hospital, Hyderabad, Pakistan (22.7%) [28], and a Tertiary Care Hospital, Wollo, Ethiopia (20.8%) [20]. The finding was much higher compared with a study conducted in the Felege Hiwot Hospital, Bahirdar, Ethiopia (2.4%) [19]. The higher rate of BSIs in this study might be due to the inclusion of patients from varying admission wards that increase the likelihood of getting an exogenous and endogenous infection of the bloodstream, while the previous study was limited to the intensive care unit and postoperative infections.

The most frequent bacteria causing NIs were *S. aureus* (18.5%) and *E. coli* (16.7%). The preponderance of *S. aureus* in this study was in agreement with a study conducted in Bahidar, Ethiopia [19] and other countries [1, 27]. This might be due to its association with the endogenous source as the organism is a member of the skin and nasal flora of the patients [23], cross-contamination from the hospital environment, surgical instruments, or hands of the health professionals [12, 29]. The second most occurrences of *E. coli* in this study might also be due to the profound influence of endogenous contamination from the bowel, hollow muscular organ of patients, and transfer from an object to the human in a hospital setting resisting common antiseptics [23, 30].

While most resistant Gram-negative and positive bacteria in this study were consistent with other findings [4, 5, 20], the resistance profile of certain strains to commonly used antimicrobials for the treatment of bacteria causing NIs was alarming. The higher prevalence of resistance observed in *S. aureus* to a number of antimicrobials (80% to each of chloramphenicol and erythromycin and 70% to each of cephalexin and tetracycline) is of a particular concern. A similar finding was reported in a tertiary hospital, Ethiopia [20], and other countries over the two decades [1, 31]. Moreover, the resistance of *S. aureus* to methicillin (88.9%) in our study was higher compared with other studies conducted elsewhere: Tertiary Care Hospital, Pakistan (64.1%) [32], Gauteng Academic Hospitals, South Africa (36%) [33], and Canadian Hospital, Canada (26%) [13]. The high rate of MRSA observed in this study might be attributed to the lack of molecular assay targeting the mecA gene [34]. We used PBP2a-agglutination test, which was the far inferior method as compared to molecular assay, to confirm MRSA. It could also be due to the incorporation of varying clinical specimens, high turnover of health professionals, low specialist staffing, high patient load, and poor prevention measures, which facilitate the circulation of resistant bacterial strains.

The isolation of MDR bacteria (42.6%) in this study was lower compared to a study conducted in Zahedan, Iran (95%) [14], Ghana (70%) [35], and Kampala, Uganda (58%) [36]. Although the rate of MDR in this study was low, it has serious implications on modern medicine and patient care outcomes. The low rate of MDR in this study might be due to lack of investigation of anaerobic bacterial NIs.

This study has several limitations. First, it was a single hospital-based study. Second, patients were not followed after discharge due to difficulties in follow up and communication. Third, anaerobic bacterial NIs were not investigated due to limited laboratory facilities. These limitations are likely to underestimate the true prevalence of NIs, and hence, the results may not be applicable to other hospitals.

## 5. Conclusions

In conclusion, the prevalence of culture-confirmed bacterial NIs in this study was comparable with other similar study findings. The most common infections were surgical site and bloodstream. *S. aureus*, *E. coli*, and *S. pneumoniae* were the most frequent causes of NIs. Most of the isolates were resistant to commonly used antimicrobials in the testing panel. The treatment and management of bacterial NIs need to be supported by culture isolation and antimicrobial susceptibility testing. Ciprofloxacin, ceftriaxone, and gentamycin should be used for the treatment of NIs when empiric treatment is unavoidable. Future studies are recommended to measure the true prevalence of NIs and antimicrobial resistance by including district hospitals, discharged patients, and communities.

## Figures and Tables

**Table 1:** Characteristics of NI-suspected patients at Hiwot Fana Specialized University Hospital, Eastern Ethiopia, March 2017 to July 2017.

Characteristics	Total (%)
Sex	
Male	171 (43.4)
Female	223 (56.6)

Age group (in years)	
Less than 10	123 (31.2)
10–19	39 (9.9)
20–29	89 (22.6)
30–39	68 (17.3)
40–49	37 (9.4)
More than 49	37 (9.4)

Admission wards	
Medical	102 (25.9)
Obstetrics/Gynecology	103 (26.1)
Pediatric	90 (22.9)
Surgical	99 (25.1)

Previous history of admission	
Yes	54 (13.7)
No	340 (86.3)

Length of hospital stay	
<4 days	142 (36)
4 to 7 days	147 (37.3)
>7 days	105 (26.7)

**Table 2:** Distribution of bacterial agents isolated from patients with NIs admitted at wards of Hiwot Fana Specialized University Hospital, Eastern Ethiopia, March 2017 to July 2017.

Bacterial isolates	Surgical site no. (%)	Urinary tract no. (%)	Respiratory tract no. (%)	Bloodstream no. (%)	Total (%)
*E. coli*	2 (11.8)	4 (36.3)	0 (0)	3 (21.4)	9 (16.7)
*Klebsiella* spp.	0 (0)	1 (9.1)	3 (25)	0 (0)	4 (7.4)
*P. vulgaris*	2 (11.8)	1 (9.1)	0 (0)	0 (0)	3 (5.5)
*P. aeruginosa*	3 (17.6)	0 (0)	2 (16.7)	1 (7.1)	6 (11.1)
*Proteus* spp.	0 (0)	2 (18.2)	0 (0)	0 (0)	2 (3.7)
*S. aureus*	5 (29.4)	1 (9.1)	0 (0)	4 (28.6)	10 (18.5)
CoNS	3 (17.6)	0 (0)	0 (0)	2 (14.3)	5 (9.3)
*S. pneumoniae*	0 (0)	0 (0)	5 (41.6)	3 (21.4)	8 (14.8)
*Streptococcus* spp.	0 (0)	0 (0)	2 (16.7)	0 (0)	2 (3.7)
*Enterococcus* spp.	2 (11.8)	2 (18.2)	0 (0)	1 (7.1)	5 (9.3)
Total	17 (31.5)	11 (20.4)	12 (22.2)	14 (25.9)	54 (100)

**Table 3:** Antimicrobial susceptibility pattern of Gram-positive bacteria isolated from NI-suspected patients at Hiwot Fana Specialized University Hospital, Eastern Ethiopia, March 2017 to July 2017.

Bacterial species	Total isolates	Pattern	Antimicrobial susceptibility, *no.* (%)							
			C	CIP	CL	E	CRO	GN	P	TE
*S. aureus*	10	S	2 (20)	8 (80)	3 (30)	2 (20)	5 (50)	7 (70)	4 (40)	3 (30)
		R	8 (80)	2 (20)	7 (70)	8 (80)	5 (50)	3 (30)	6 (60)	7 (70)

CoNS	5	S	3 (60)	3 (60)	4 (80)	2 (40)	5 (100)	4 (80)	4 (80)	2 (40)
		R	2 (40)	2 (40)	1 (20)	3 (60)	0 (0)	1 (20)	1 (20)	3 (60)

*S. pneumoniae*	8	S	7 (87.5)	6 (75)	5 (62.5)	2 (25)	5 (62.5)	4 (50)	3 (37.5)	2 (25)
		R	1 (12.5)	2 (25)	3 (37.5)	6 (75)	3 (37.5)	4 (50)	5 (62.5)	6 (75)

*Streptococcus* spp.	2	S	2 (100)	1 (50)	0 (0)	0 (0)	2 (100)	2 (100)	2 (100)	0 (0)
		R	0 (0)	1 (50)	2 (100)	2 (100)	0 (0)	0 (0)	0 (0)	2 (100)

*Enterococcus*	5	S	3 (60)	4 (80)	3 (60)	4 (80)	3 (60)	4 (80)	1 (20)	1 (20)
		R	2 (40)	1 (20)	2 (40)	1 (20)	2 (40)	1 (20)	4 (80)	4 (80)

Total	30	S	17 (56.7)	22 (73.3)	15 (50)	10 (32.3)	18 (60)	21 (70)	14 (46.7)	8 (26.7)
		R	13 (43.3)	8 (26.7)	15 (50)	20 (66.7)	12 (40)	9 (30)	16 (53.3)	22 (73.3)

S: sensitive; R: resistance; C: chloramphenicol; CIP: ciprofloxacin; CL: cephalexin; E: erythromycin; CRO: ceftriaxone; GN: gentamycin; P: penicillin; TE: tetracycline.

**Table 4:** Antimicrobial susceptibility pattern of Gram-negative bacteria isolated from NI-suspected patients at Hiwot Fana Specialized University Hospital, Eastern Ethiopia, March 2017 to July 2017.

Bacterial species	Total isolates	Pattern	Antimicrobial susceptibility, *no.* (%)					
			C	CAZ	CIP	CL	CRO	GN
*E. coli*	9	S	3 (33.3)	2 (22.2)	9 (100)	7 (77.8)	7 (77.8)	6 (66.7)
		R	6 (66.7)	7 (77.8)	0 (0)	2 (22.2)	2 (22.2)	3 (33.3)

*Klebsiella* spp.	4	S	2 (50)	1 (25)	4 (100)	3 (75)	3 (75)	2 (50)
		R	2 (50)	3 (75)	0 (0)	1 (25)	1 (25)	2 (50)

*P. vulgaris*	3	S	1 (33.3)	2 (66.7)	2 (66.7)	2 (66.7)	2 (66.7)	2 (66.7)
		R	2 (66.7)	1 (33.3)	1 (33.3)	1 (33.3)	1 (33.3)	1 (33.3)

*P. aeruginosa*	6	S	2 (33.3)	1 (16.3)	4 (66.7)	1 (16.3)	3 (50)	4 (66.7)
		R	4 (66.7)	5 (83.7)	2 (33.3)	5 (83.7)	3 (50)	2 (33.3)

*Proteus* spp.	2	S	1 (50)	2 (100)	2 (100)	0 (0)	2 (100)	1 (50)
		R	1 (50)	0 (0)	0 (0)	2 (100)	0 (0)	1 (50)

Total	24	S	9 (37.5)	8 (33.3)	21 (78.5)	13 (54.2)	17 (70.8)	16 (66.7)
		R	15 (66.5)	16 (66.7)	3 (12.5)	11 (45.8)	7 (29.2)	8 (33.3)

S: sensitive; R: resistance; C: chloramphenicol; CAZ: ceftazidime; CIP: ciprofloxacin; CL: cephalexin; CRO: ceftriaxone; GN: gentamycin.

## Data Availability

The SPSS data used to support the findings of this study are available from the corresponding author upon request.
